# Performance of D-dimer for predicting sepsis mortality in the intensive care unit

**DOI:** 10.11613/BM.2021.020709

**Published:** 2021-06-15

**Authors:** Yan-Qiu Han, Li Yan, Lei Zhang, Pei-Heng Ouyang, Peng Li, Giuseppe Lippi, Zhi-De Hu

**Affiliations:** 1 Department of Laboratory Medicine, The Affiliated Hospital of Inner Mongolia Medical University, Hohhot, China; 2 Department of Respiratory and Critical Care Medicine, The Affiliated Hospital of Inner Mongolia Medical University, Hohhot, China; 3 Section of Clinical Biochemistry, University of Verona, Verona, Italy

**Keywords:** sepsis, fibrin/fibrinogen degradation products, intensive care units, treatment outcome, disseminated intravascular coagulation

## Abstract

**Introduction:**

The prognostic value of D-dimer (DD) in sepsis remains controversial. This study aimed to investigate the performance of DD for predicting sepsis mortality in the hospital and for identifying its potential correlates.

**Materials and methods:**

The clinical and laboratory data of adult sepsis patients were extracted from the Medical Information Mart for Intensive Care III (MIMIC III, v1.4) database using the structured query language (SQL). The database contains critical illness admitted to the intensive care unit at Beth Israel Deaconess Medical Center between June 2001 and October 2012. The association between DD and mortality was investigated with receiver operating characteristic (ROC) curve, restricted cubic spline and logistic regression analysis. Subgroup analysis was also used for identifying DD correlates.

**Results:**

The study population consisted of 358 sepsis patients. Those who died during hospital stay (N = 160) had significantly higher DD values than those who survived (N = 198). The area under the ROC curve (AUC) of DD was 0.59 (P < 0.010). In subgroup analysis, white blood cell (WBC) count > 18 x10^9^/L and vasopressor therapy significantly decreased DD diagnostic performance. Categorical DD value was independently associated with hospital mortality after sequential organ failure score (SOFA) and blood lactate adjustment. Restricted cubic spline analysis revealed a U-shape relationship between DD and in-hospital mortality.

**Discussion:**

We conclude that the accuracy of DD for predicting in-hospital sepsis mortality depends on WBC count and vasopressor therapy. Both low and extremely elevated DD values are associated with higher risk of death.

## Introduction

Sepsis is a major cause of morbidity and mortality in patients admitted to the intensive care unit (ICU). According to the Third International Consensus Definitions for Sepsis and Septic Shock (Sepsis-3) definition, sepsis is now considered as a “life-threatening organ or system dysfunction, caused by a dysregulated host response to infection” (*1*). Among all types of biological dysregulations, haemostasis derangement is very frequent in septic patients (*2*). Disseminated intravascular coagulation (DIC) is a form of systemic blood clotting, mostly characterized by widespread activation of both platelets and coagulation cascade, leading to massive thrombin generation, eventually evolving towards diffuse thrombosis and multiple organ failure (MOF) (*3*). Recent evidence suggests that the prevalence of acute DIC in patients with sepsis may be comprised between 25-50%, and its onset enormously magnifies the risk of death (*4*-*7*).

Unlike fibrin/fibrinogen degradation products (FDP), D-dimer (DD) is a specific degradation product of stabilized fibrin (*8*). Therefore, the presence of increased value of this biomarker reflects both thrombin generation, as well as fibrinolytic degradation. Several studies have addressed the potential prognostic value of DD in sepsis over the past decades, providing quite inconsistent or even controversial evidence. Although increased DD values have been associated with worse clinical outcomes in some studies, others failed to confirm such findings, revealing that the prognostic value of DD may be modest or poor in sepsis patients (*9*-*14*). Notably, a recent study has also demonstrated that sepsis patients with DD values within the normal reference range had a nearly 4-fold higher risk of dying than those with DD concentration modestly or markedly increased (*15*). Since confirmatory evidence would be needed to define the putative prognostic significance of measuring DD in patients with sepsis, as well as its potential clinical and laboratory correlates in this setting, we performed a retrospective study aimed to address the diagnostic performance of this biomarker in predicting in-hospital sepsis mortality.

## Materials and methods

### Study design

This retrospective study was based on an analysis of the Medical Information Mart for Intensive Care III (MIMIC III, v1.4) database. The structured query language (SQL) was used to extract data from MIMIC III, which is a freely accessible clinical database, encompassing a total number of 46,520 patients and 58,976 ICU admissions (*16*, *17*). All patients were admitted to the ICU at Beth Israel Deaconess Medical Center between June 2001 and October 2012. The database has been originally developed by the Massachusetts Institute of Technology (MIT), and all included patients have been de-identified to preserve their privacy. After having passed a mandatory examination on the website of the National Institutes Health (NIH), an author of this manuscript was allowed to obtain data from MIMIC III for research purposes. Informed consent is inherently waived due to data availability in form of the public database.

### Study population

The inclusion criteria of this study were as follows: (i) age > 18 years, (ii) definitive diagnosis of sepsis based on the International Classification of Diseases (ICD 9) code 995.92 (sepsis) and 785.52 (septic shock), which are currently recommended by the Sepsis-3, and (iii) at least one DD value measured within 24 hours after ICU admission (in patients with many sequential DD values available, the first one measured at ICU admission was selected). The plasma DD was assayed using HemosIL D-Dimer HS 500 kit on the Instrumentation Laboratory ACL TOP automated analyzer (Instrumentation Laboratory, Lexington, USA).

The following additional information was then extracted from the database: demographic characteristics, results of laboratory testing and blood gas analysis, severity scores, treatment modalities, comorbidity, length of stay in the ICU, and outcome (*i.e.*, in-hospital death). All laboratory tests, except DD, were collected in the first 24 hours of patient admission. The sequential organ failure score (SOFA) and simplified acute physiology score (SAPSII) scores were calculated as previously described (*18*, *19*).

### Statistical analysis

The Kolmogorov-Smirnov test was used to verify the normal distribution of continuous data. Normal distributed continuous variables were expressed as mean and standard deviation (SD), and comparisons were made using Student’s t test. Skewed data were instead expressed as median (and interquartile range; IQR) and comparisons were made using the Mann-Whitney U test. Categorical data comparison was performed with the Chi-square test. The diagnostic accuracy of DD value for predicting in-hospital mortality was analysed with receiver operating characteristic (ROC) curve analysis, by calculation of the area under the curve (AUC) and its 95% confidence interval (95% CI), and with multivariate logistic regression models. A restricted cubic spline was used to analyse the relationship between DD and in-hospital mortality. All analyses were performed with R (version 3.5.0) and statistical significance was set at P<0.05.

## Results

### Characteristics of the study population

**Figure 1:**
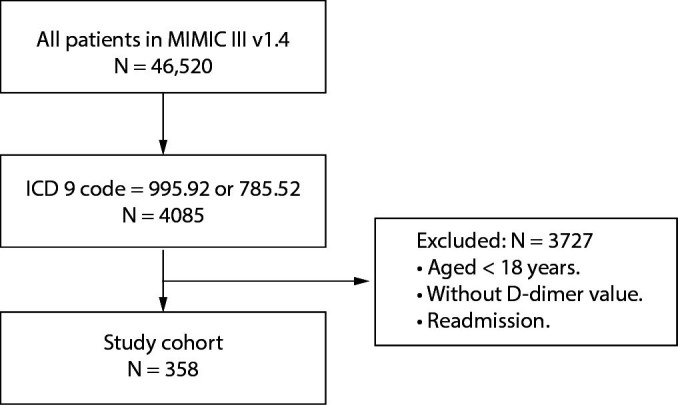
Flowchart of subjects’ selection. MIMIC III – Medical Information Mart for Intensive Care III. ICD 9 – International Classification of Diseases.

The flowchart illustrating the population study selection is shown in Figure 1. A total of 358 patients could be included in this study, and their clinical and laboratory characteristics are summarized in Table 1. A 160/358 patients (44.7%) died during hospitalization. Patients who died had significantly lower values of white blood cell (WBC) count, haemoglobin, platelets, and albumin. The values of bilirubin, blood lactate, activated partial thromboplastin time (APTT), prothrombin time (PT), international normalized ratio (INR), SOFA, and SAPSII scores were also found to be significantly higher in patients who died. A larger prevalence of liver disease and coagulopathy could also be observed in patients who died, whilst the rate of patients who received renal replacement therapy (RRT), mechanical ventilation, and vasopressor therapy was also higher in patients who died.

**Table 1:** Summary of the cohort

**Variables**	**Total** **N = 358**	**Alive** **N = 198**	**Dead** **N = 160**	**P**
Age, years	64 (52-78)	64 (49-78)	64 (54-76)	NS
Male, N (%)	196 (55)	100 (51)	96 (60)	NS
White, N (%)	244 (68)	133 (67)	111 (69)	NS
Emergency, N (%)	346 (97)	191 (96)	155 (97)	NS
WBC, x10^9^/L	16.0 (8.6-24.0)	17.7 (11.2-26.1)	13.5 (5.8-21.1)	< 0.001
Haemoglobin, g/L	113 (101-127)	116 (105-131)	110 (98-123)	0.002
Platelet, x10^9^/L	160 (88-254)	182 (122-268)	128 (70-225)	< 0.001
Albumin, g/L	28 (23-31)	28 (24-32)	27 (23-31)	0.049
Bilirubin, μmol/L	22 (9-56)	15 (9-43)	33 (14-75)	< 0.001
Creatinine, μmol/L	159 (106-248)	159 (97-265)	168 (115-239)	NS
Lactate, mmol/L	3.9 (2.2-6.7)	3.3 (2.0-5.0)	5.2 (2.8-8.7)	< 0.001
APTT, s	45.6 (35.3-68.4)	40.6 (33.6-58.4)	51.0 (39.7-74.0)	< 0.001
INR	1.9 (1.5-3.1)	1.7 (1.4-2.3)	2.3 (1.7-3.7)	< 0.001
PT, s	19.0 (16.1-25.2)	17.3 (15.6-21.3)	20.8 (17.2-30.3)	< 0.001
DD, μg/L	4098 (1683-7828)	3393 (1501-6835)	4580 (2039-8311)	0.006
SOFA	9 (7-12)	8 (5-11)	11 (8-13)	< 0.001
SAPSII	51.44 ± 16.85	45.28 ± 15.45	59.07 ± 15.35	< 0.001
CHF, N (%)	135 (38)	72 (36)	63 (39)	NS
Cardiac arrhythmias, N (%)	139 (39)	79 (40)	60 (38)	NS
Hypertension, N (%)	153 (43)	84 (42)	69 (43)	NS
Chronic pulmonary, N (%)	63 (18)	35 (18)	28 (18)	NS
Renal failure, N (%)	71 (20)	37 (19)	34 (21)	NS
Liver disease, N (%)	110 (31)	40 (20)	70 (44)	< 0.001
Coagulopathy, N (%)	198 (55)	99 (50)	99 (62)	0.032
SO2, %	95 (91-98)	94 (91-97)	95 (93-98)	NS
SPO2, %	98 (95-100)	98 (95-100)	98 (95-99)	NS
PCO2, kPa	4.8 (4.0-6.0)	4.8 (4.1-5.9)	4.9 (3.6-6.1)	NS
RRT, N (%)	40 (11)	12 (6)	28 (18)	0.001
Vasopressors, N (%)	293 (82)	149 (75)	144 (90)	< 0.001
Ventilation, N (%)	249 (70)	116 (59)	133 (83)	< 0.001
Days of ICU, days	4 (2-9)	5 (3-9)	3 (1-8)	< 0.001
Days of hospital, days	9 (5-16)	10 (7-18)	5 (2-12)	< 0.001
Severe sepsis, N (%)	328 (92)	184 (93)	144 (90)	NS
Septic shock, N (%)	259 (72)	144 (73)	115 (72)	NS
Data with normal distribution were expressed as mean and standard deviation and compared by Student’s t test. Skewed data were expressed as median (interquartile range) and compared by Mann-Whitney U test. Categorical data were expressed as absolute number (percentage) and compared by Chi-square test. WBC – white blood cell. APTT – activated partial thromboplastin time. PT – prothrombin time. INR – international normalized ratio. DD – D-dimer. SOFA – sequential organ failure score. SAPSII – simplified acute physiology score. CHF – congestive heart failure. SO2 – oxygen saturation. SPO2 – pulse oximetry. PCO2 – partial pressure of carbon dioxide. RRT – renal replacement therapy. ICU – intensive care unit. NS – non-significant.				

### Performance of DD for predicting in-hospital mortality

**Figure 2:**
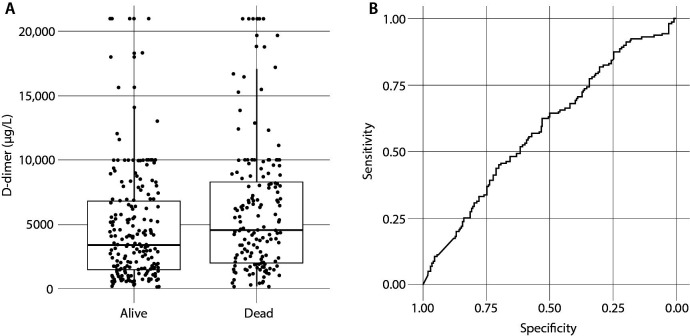
D-dimer predicts hospital mortality. A) Patients without hospital mortality had lower DD than those with hospital mortality. B) Receiver operating characteristic curve of DD for hospital mortality. DD - D-dimer.

**Figure 3:**
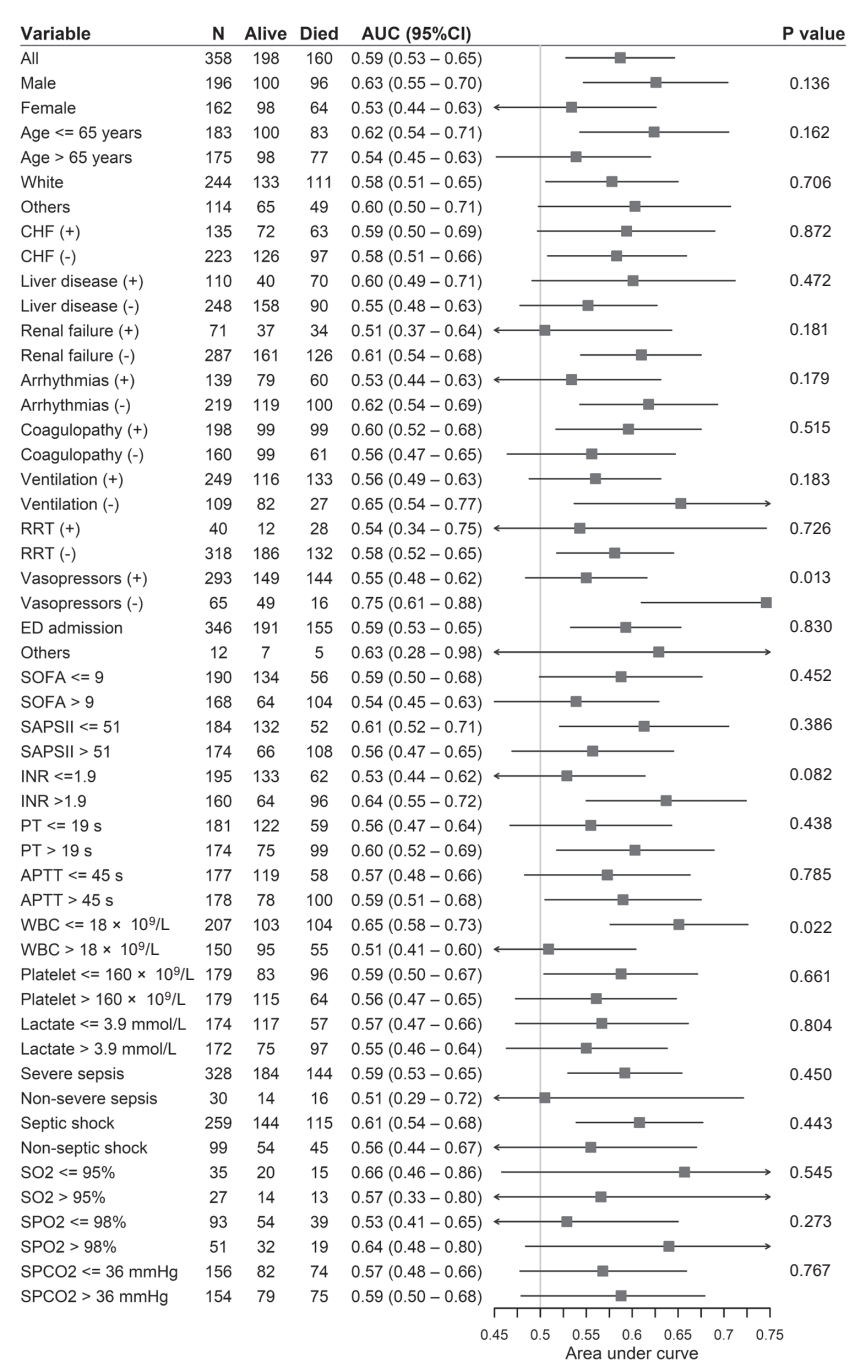
Forest plot display the area under curve of DD for hospital mortality. CHF – congestive heart failure. RRT – renal replacement therapy. ED – Emergency Department. SOFA – sequential organ failure score. SAPSII – simplified acute physiology score. INR – international normalized ratio. PT – prothrombin time. APTT – activated partial thromboplastin time. WBC – white blood cell. SO2 – oxygen saturation. SPO2 – pulse oximetry. PCO2 – partial pressure of carbon dioxide.

D-dimer values were found to be significantly higher in patients who died during hospital stay than in those who survived (Figure 2A). The ROC curve analysis, performed for evaluating the accuracy of DD values for predicting in-hospital mortality is shown in Figure 2B. The AUC of DD was 0.59 (95% CI, 0.53-0.65; P < 0.010). The diagnostic accuracy of DD for predicting in-hospital mortality was also studied in some subgroups of patients, clustered according to their clinical characteristics, as shown in Figure 3. The AUC of DD in patients without vasopressor therapy was significantly higher than that in those undergoing treatment with these agents (0.75 *vs.* 0.55; P = 0.013). The predictive accuracy of DD was also higher in patients with WBC < 18 x10^9^/L than in those with WBC > 18 x10^9^/L (0.65 *vs.* 0.51; P = 0.022).

Since a previous study reported that sepsis patients with normal DD concentration had worse outcome than those with increased values, we further compared in-hospital mortality between patients with normal (*i.e.*, < 500 μg/L), increased (*i.e.*, between 500 and 4000 μg/L) and extremely high (*i.e.*, > 4000 μg/L) DD values (*15*). The corresponding mortality rates in patients with normal, increased and extremely increased DD values were 0.60 (9/15), 37.3% (60/161) and 50.0% (91/182), exhibiting a statistical significance for trend (P = 0.029 by Chi-square test). Notably, the risk of death was also higher in patients with markedly increased DD values (*i.e.*, > 4000 μg/L) than in those with modestly elevated concentrations (*i.e.*, 500-4000 μg/L, odds ratio (OD): 1.68; 95% CI: 1.09-2.59; P = 0.020).

**Figure 4:**
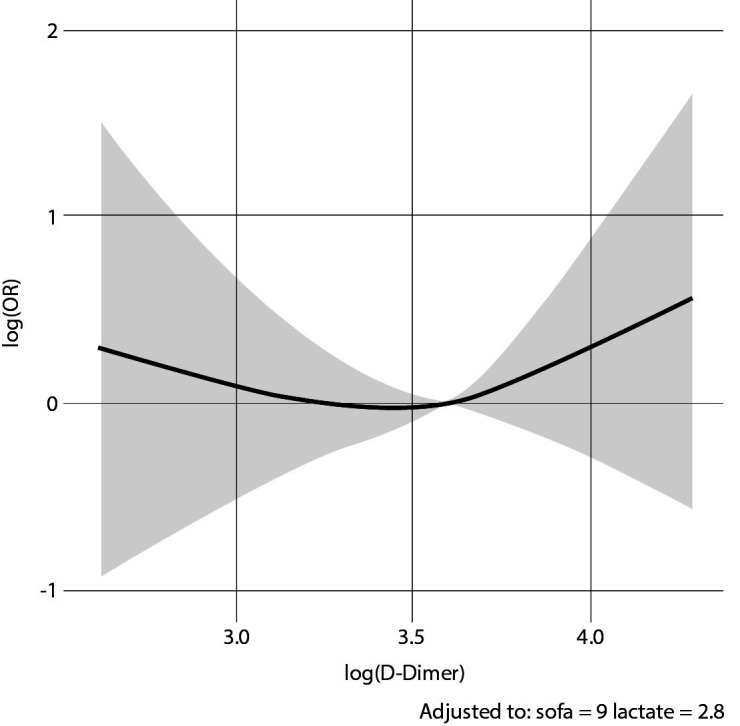
Restricted cubic spline reveals a U-shape relationship between DD and in-hospital mortality. OD – odds ratio. SOFA – sequential organ failure score.

In addition, we used the restricted cubic spline method to portray the relationship between DD and in-hospital mortality risk. As shown in Figure 4, a clear U-shape relationship could be observed between DD and the risk of in-hospital death. The DD concentration with the lowest risk of in-hospital mortality was found to be approximately 2700 μg/L.

### Multivariate analysis

The results of logistic regression models carried out for evaluating the diagnostic accuracy of DD values in predicting in-hospital mortality are summarized in Table 2. Since some laboratory tests, especially platelet count and bilirubin, are included in the SOFA score, multiple adjustments were only carried out for SOFA and natural logarithm transformed lactate in our multivariate analysis. According to this analysis, natural logarithm transformed DD value was associated with in-hospital mortality in univariate statistics, whilst such association was lost in multivariate analysis. Next, we transformed DD as a categorical variable (*i.e.*, < 500 μg/L, 500–4000 μg/L and > 4000 μg/L) (*15*). To facilitate results presentation, we set DD values 500-4000 μg/L as a reference. As shown in Table 2, the ODs of high DD (> 4000 μg/L) and low DD (< 500 μg/L) were larger than 1, suggesting that both increased and decreased DD would be associated with a higher risk of in-hospital mortality.

**Table 2:** Prognostic value of DD with logistic regression models

	**Univariate**		**Multivariate**	
	OR (95% CI)	P	OR (95% CI)	P
Ln (DD)	1.34 (1.09–1.66)	0.010	1.00 (0.79–1.29)	NS
< 500 μg/L	2.53 (0.87–7.86)	NS	4.87 (1.46–17.23)	0.010
500–4000 μg/L	1 (Reference)	NA	1 (Reference)	NA
> 4000 μg/L	1.68 (1.10–2.60)	0.020	1.05 (0.64–1.74)	NS
OR – odds ratio. CI – confidence interval. NS – non-significant. NA – not applicable. Factors adjusted in multivariable logistic regression model are SOFA score and natural logarithm transformed lactate. DD - D-dimer.				

## Discussion

The identification of prognostic factors remains crucial in the management of sepsis, whereby treatment selection is currently considered the largest variable influencing patient outcomes. Some interesting findings have emerged from this study. First, we observed a U-shape relationship between DD values and in-hospital mortality in sepsis patients. The prognostic value of DD was found to be independent from the SOFA score, a widely used tool for predicting the outcome of sepsis. We then observed that the diagnostic accuracy of DD for predicting in-hospital sepsis mortality is largely dependent on WBC count and vasopressor therapy, whereby the prognostic value of this biomarker was higher in patients who did not receive vasopressors and with WBC count < 18 x 10^9^/L. The results of this study hence seemingly suggest that more intensive treatment strategies would be necessary for patients with non-diagnostic (*i.e.*, lower than the cut-off) or extremely elevated DD values. The most important conclusion emerging from this finding is that diagnosing DIC in sepsis patients using the criteria established by the International Society on Thrombosis and Hemostasis (ISTH) may be misleading, as suggested by Semeraro and colleagues since we also observed that patients with non-diagnostic DD concentration may have up to 80% higher risk of death compared to those with values exceeding the diagnostic threshold (*15*, *20*). Considering the retrospective nature of this study, characterized by modest sample size, future studies with prospective design and large sample size would be needed to validate our findings as well as those earlier published by Semeraro *et al.* (*15*).

Some hypotheses can also be brought in support of our findings. For example, the risk of mortality may be substantially enhanced in patients with prevalently thrombotic DIC subtypes, where fibrinolysis inhibition is exceptionally high, thus limiting thrombus (and fibrin) degradation, increasing disseminated thrombotic injuries, and ultimately preventing the increase of DD values in the bloodstream (*15*).

This study has strength compared with previous investigations. First, the sample size (N = 358) was higher than that of previous studies which have also attempted to define the prognostic value of DD in sepsis. Then, many earlier studies did not adequately address the potential clinical and laboratory correlates of DD, which can ultimately influence its prognostic value. Notably, in the subgroup analysis carried out in our study, WBC count and vasopressor therapy were found to have an important impact in influencing the prognostic value of DD. The majority of previous studies also hypothesized the existence of a linear relationship between DD and sepsis mortality, whereby DD have been frequently associated with worse outcome. Nevertheless, in our additional analysis, where patients were stratified according to their DD values upon ICU admission as being normal (< 500 μg/L), modestly increased (500-4000 μg/L) or markedly elevated (> 4000 μg/L), we found that patients with very low DD values paradoxically had the highest overall risk of death, up to 60%.

These original findings can partially explain the heterogeneous evidence that emerged from previous studies which have also addressed the prognostic value of DD in sepsis. The vast majority of these previous investigations compared DD values in patients who died or survived, and then assessed its prognostic accuracy with ROC curve analysis (*9*-*14*). These two statistical methods would only be useful for biomarkers displaying a linear relationship with clinical outcome, since DD values distribution in cohort studies may have a remarkable influence on results. Moreover, the heterogeneous findings reported by previous studies may also be attributed to a lack of adjustment of final mortality analysis for WBC count and vasopressor treatment, which were instead important determinant of DD predictive accuracy in our study.

This study has some limitations, the first of which is the retrospective design. Then, DD value was unavailable for some sepsis patients within the first 24 hours upon ICU admission, and these subjects were hence excluded from our study. Finally, two ICD 9 codes (995.92 and 785.52) were used for identifying sepsis patients, which are highly specific, but less sensitive, for sepsis (*21*).

We could hence conclude that DD value may be an independent prognostic factor for in-hospital mortality in sepsis patients, and its prognostic accuracy is influenced by vasopressor therapy and WBC count. According to our data, a U-shape relationship can be postulated between DD values and in-hospital mortality. Both normal (*i.e.*, < 500 μg/L) and extremely elevated (*i.e.*, > 4000 μg/L) concentrations were associated with higher in-hospital mortality. Considering that this is a retrospective single center study, further prospective, multicenter studies with a large sample size and a broader spectrum of sepsis patients would be needed to validate our findings.
